# Long time blood-transfusion trend in a European general hospital

**DOI:** 10.17179/excli2020-2526

**Published:** 2020-06-19

**Authors:** Dietmar Enko, Markus Herrmann, Andreas Baranyi, Wolfgang J. Schnedl, Gabriele Halwachs-Baumann

**Affiliations:** 1 Clinical Institute of Medical and Chemical Laboratory Diagnostics, Medical University of Graz, Graz, Austria; 2 Institute of Clinical Chemistry and Laboratory Medicine, General Hospital Hochsteiermark, Austria; 3 Institute of Clinical Chemistry and Laboratory Medicine, General Hospital Steyr, Austria; 4 Department of Psychiatry and Psychotherapeutic Medicine, Medical University of Graz, Graz, Austria; 5 Practice for General Internal Medicine, Bruck/Mur, Austria

## ⁯

***Dear Editor,***

In recent years, concerns about transfusion-related risk factors, high blood acquisition costs, and blood shortages have gained substantial interest of hospitals in finding possible ways to decrease blood transfusions. Based on the knowledge of the side effects of allogenic blood transfusions, patient blood management (PBM) developed into an evidence-based, interdisciplinary clinical approach, which aims to reduce unnecessary transfusions (Murphy and Goodnough, 2015[19]).

PBM programs are based on three pillars, namely optimizing anemia management, minimizing blood loss and bleeding, and harnessing patient's physiological reserve of anemia (Leahy et al., 2014[13]). Currently, a broad variety of different guidelines on best transfusion practices and consensus statements of perioperative anemia management are available (Meybohm et al., 2017[15]; Carson et al., 2016[4]; Muñoz et al., 2017[17], 2018[18]). Since anemia is a major predicting factor in requiring perioperative allogenic blood transfusions (Walsh et al., 2012[25]; Wong et al., 2015[26]), strategies for preoperative diagnosis and treatment of this risk factor are strictly recommended (Meybohm et al., 2017[16]).

In our hospital, which covers a catchment area of approximately 300.000 inhabitants, we implemented an algorithm of preoperative anemia management as part of a PBM program in October 2011 (Enko et al., 2013[6]). Since that date, anemic individuals with elective surgery obtain an adequate preoperative anemia classification and treatment with erythropoietin and intravenous iron (Enko et al., 2013[6]). A recent study comprising seven European university hospitals demonstrated that the implementation of PBM activities is highly variable (Bruun et al., 2016[3]). Perioperative anemia management programs are not generally and homogeneously implemented between these hospitals of different European countries (Manzini et al., 2018[14]). 

It has also been shown that transfusion practices vary highly among hospitals even in a single country (Gombotz et al., 2007[8]). Only sparse data are available describing the long-time transfusion trends in Austrian hospitals. Since continuous monitoring of the transfusion activities and the implementation of optimized transfusion strategies may improve daily transfusion practice and decrease the need of blood, it would be of great interest to assess such data for hospitals in each country. 

Therefore, this study was conducted to analyze a 12-year trend of transfused red blood cell (RBC), platelet and plasma units comprising a 6-year period before and after implementation of an anemia management program in a general hospital in Austria.

A total of 49,142 RBC units were transfused between 2006 - 2017 in our hospital. As shown in Table 1(Tab. 1), a distinct decrease of transfused (22,745 vs. 26,397, -13.8 %) RBC units was observed in the period after the implementation of the algorithm-guided anemia management program (2012 - 2017) compared to the period before PBM initiation (2006 - 2011).

**Figure 1:**
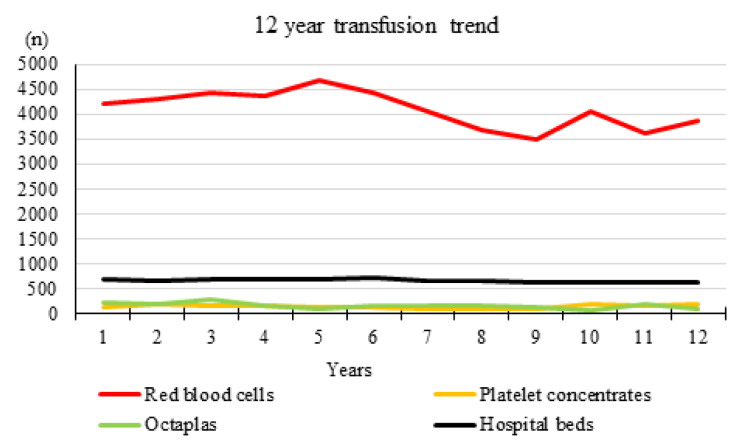
Twelve-year (2006 - 2017) transfusion trend of RBC, platelet and plasma concentrates in an Austrian general hospital with an average number of 657 hospital beds during the investigated time period

**Figure 2:**
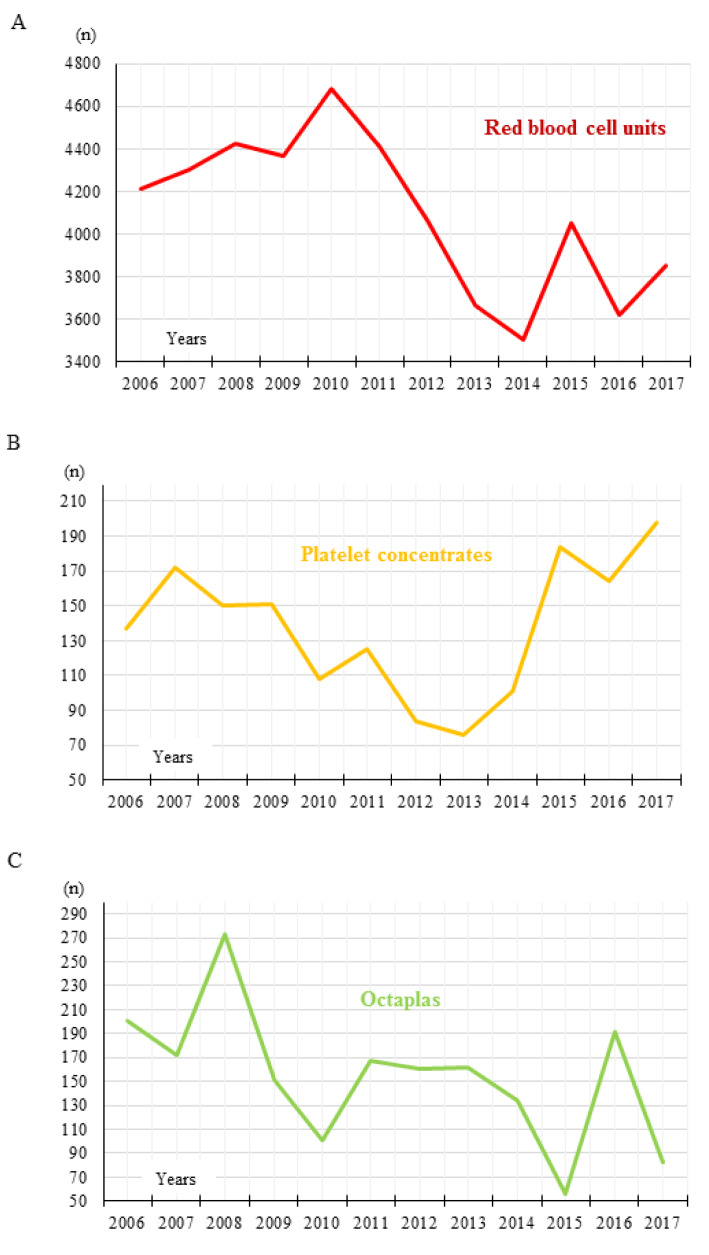
Transfused RBC, platelet and plasma units. Detailed pattern of (A) RBC unit, (B) platelet concentrate, and (C) Octaplas use between 2006 and 2017

Figure 1(Fig. 1) gives an overview of the RBC, platelet and plasma use over all 12 evaluated years. The detailed pattern of transfused RBC, platelet and plasma (Octaplas) units is shown in Figure 2(Fig. 2). The average plasma unit use decreased distinctly after PBM implementation compared to the period before (787 vs. 1065 units, - 26.1 %). A slight decrease of platelet concentrate use (807 vs. 843 units, - 4.3 %) was observed, only. Platelet concentrates showed a wide range of deviation with a maximum of 198 transfused units in 2017 and a minimum of 76 transfused units in 2013. In the present study, we evaluated a 12-year trend on RBC, platelet and plasma unit transfusion practice at an Austrian general hospital comparing a six-year period before and a six-year period after implementation of an anemia management program. We observed a RBC transfusion reduction of -13.8 % after PBM initiation in 2011. The plasma and platelet unit reduction were -26.1 and -4.3 %.

In comparison, a previously published retrospective study, which analyzed RBC, platelet and plasma utilization after implementing PBM initiatives in seven American hospitals between 2007 and 2015, showed a 29.9 %, a 25.7 %, and a 24.8 % reduction in transfused RBC, platelet and plasma units (Verdecchia et al., 2016[23]). Another retrospective work reported significant reduction of RBC transfusions in an American general hospital after implementation of a restrictive transfusion policy (Yerrabothala et al., 2014[28]). Several recent studies demonstrate that the total RBC, platelet and plasma units transfused in hospitals continuously declined within the last decade (Ellingson et al., 2017[5]; Laurén et al., 2019[12]; Nordestgaard et al., 2020[20]; Kimball et al., 2019[11]).

Nevertheless, it still exists a wide variation in transfusion practices after non-cardiac and cardiac surgery between different institutions (Abdelsattar et al., 2015[1]; Qian et al., 2013[21]; Rogers et al., 2009[22]). Both Austrian benchmark studies for blood utilization in elective surgery demonstrated significant variabilities in the number of perioperative RBC transfusions between the hospitals (Gombotz et al., 2007[8], 2014[9]). Substantial variations in blood loss during surgery procedures and in the utilization of PBM measures may be potential reasons for these very high intercenter differences (Gombotz et al., 2014[9]). 

Here, we implemented an algorithm-guided anemia management with erythropoietin and iron therapy to reduce allogenic RBC transfusions (Enko et al., 2013[6]). A recent meta-analysis including seven studies reported, that erythropoietin is a very effective and safe anemia management drug to reduce allogenic transfusions (Voorn et al., 2016[24]). Moreover, intravenous iron therapy was shown to be an effective tool to reduce blood transfusions, lengths of hospital stays, and costs (Froessler et al., 2018[7]; Beverina et al., 2020[2]). Evidence-based literature suggests that erythropoietin and iron therapy combined is more effective in anemia management compared to intravenous iron therapy alone (Kei et al., 2019[10]).

In addition to the implementation of an anemia management program in our hospital, we changed the RBC transfusion practice with the initiation of an educational program within the last few years. Every physician, who starts a new job at our hospital, regardless of the department, is obligated to undergo an education program, which comprises the three pillars of PBM (i.e., optimize RBC mass, minimize blood loss, manage anemia) and basics of transfusion medicine. Although education alone is often ineffective in changing behavior, internal training of clinical staff is suggested as one essential part of PBM measures in tertiary care hospitals (Leahy et al., 2014[13]; Yao et al., 2018[27]). However, further continuing PBM activities are necessary to improve daily clinical transfusion practice and to perpetuate the decreasing trend of RBC, platelet and plasma administration.

The present study has some limitations, which must be mentioned. This is a single-center study, which reflects a single-institutional experience and limits the ability to generalize the results to other institutions. Although the transfused RBC, platelet and plasma units decreased during the observation period, the appropriateness of the administered transfusions cannot be evaluated by these data. Blood transfusions were not categorized as single, 2-unit, or multiunit transfusions.

The 12-year pattern of blood use in an Austrian General Hospital investigated here showed a distinct decreasing trend of transfused RBC and plasma units. The implementation of an algorithm-guided anemia management program and the awareness of clinicians about PBM measures may have an impact of blood transfusion rates. However, further improvement activities are necessary to minimize blood transfusion in clinical practice.

For additional information see the Supplementary material.

## Conflict of interest

The authors declare that they have no conflict of interest.

## Supplementary Material

Supplementary material

## Figures and Tables

**Table 1:**
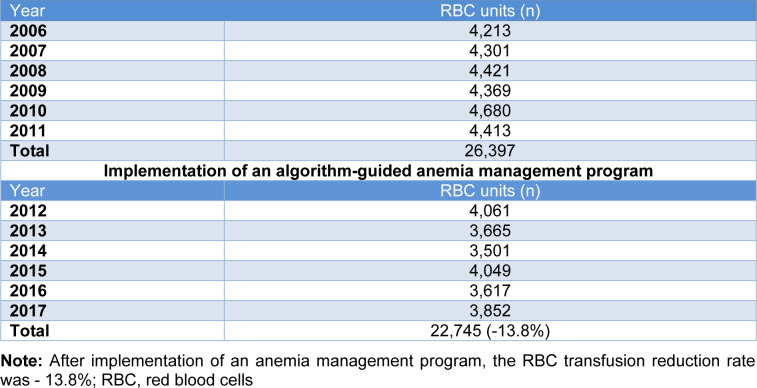
Transfused RBC units between 2006 and 2017
