# Supplementary material for: Long time blood-transfusion trend in a European general hospital
Source: EXCLI J. 2020 Jun 19;19:855–60. doi: 10.17179/excli2020-2526 (PMC7527503; doi:10.17179/excli2020-2526)
Supplement: Supplementary material [file EXCLI-19-855-s-001.pdf]

EXCLI Journal 2020;19:855-860 – ISSN 1611-2156
Received: June 09, 2020, accepted: June 16, 2020, published: June 19, 2020

Supplementary material to:

Letter to the editor:

LONG TIME BLOOD-TRANSFUSION TREND IN A EUROPEAN GENERAL HOSPITAL

Dietmar Enko[a,b,e*], Markus Herrmann[a], Andreas Baranyi[c], Wolfgang J. Schnedl[d], Gabriele Halwachs-Baumann[e]

[a] Clinical Institute of Medical and Chemical Laboratory Diagnostics, Medical University of Graz, Graz, Austria
[b] Institute of Clinical Chemistry and Laboratory Medicine, General Hospital Hochsteiermark, Austria
[c] Department of Psychiatry and Psychotherapeutic Medicine, Medical University of Graz, Graz, Austria
[d] Practice for General Internal Medicine, Bruck/Mur, Austria
[e] Institute of Clinical Chemistry and Laboratory Medicine, General Hospital Steyr, Austria

* **Corresponding author:** Dietmar Enko, Clinical Institute of Medical and Chemical Laboratory Diagnostics, Medical University of Graz, Auenbruggerplatz 15, 8036 Graz, Austria, Telephone: +43-316-385-13145, Fax: +43-316-385-13430, E-mail: enko.dietmar@gmx.at

http://dx.doi.org/10.17179/excli2020-2526

This is an Open Access article distributed under the terms of the Creative Commons Attribution License (http://creativecommons.org/licenses/by/4.0/).

MATERIALS AND METHODS

Study design

This retrospective study investigates the numbers of RBC, platelet and plasma (Octaplas, Octapharm AG, Lachen Switzerland) units, which were transfused during a twelve-year period in the General Hospital Steyr (Steyr, Austria). Blood transfusions from January 01, 2006 to December 31, 2017 were analyzed.

In October 2011, an algorithm-guided anemia management program as part of PBM was implemented in the Hospital. The trends of blood transfusions were evaluated before (2006 – 2011) and after (2012 – 2017) the initiation of this PBM program. The average number of hospital beds during this period was 657, with a minimum of 624 in 2017, and a maximum of 707 in 2011.

The study was approved by the Ethical Committee of the Johannes Kepler University Linz (Linz, Austria) (trial registration number: 1129/2018) and performed in accordance with the ethical standards of the Declaration of Helsinki. Informed consent was not required because all collected data remained anonymous.

Statistical analysis

Descriptive statistics were performed to evaluate and tabulate the RBC, platelet and plasma units transfused per year over a time-period of 12 years.
